# Do Childhood Adversities Predict Suicidality? Findings from the General Population of the Metropolitan Area of São Paulo, Brazil

**DOI:** 10.1371/journal.pone.0155639

**Published:** 2016-05-18

**Authors:** Bruno Mendonça Coêlho, Laura Helena Andrade, Guilherme Borges, Geilson Lima Santana, Maria Carmen Viana, Yuan-Pang Wang

**Affiliations:** 1 Section of Psychiatric Epidemiology – LIM-23, Department and Institute of Psychiatry, University of São Paulo Medical School, São Paulo, SP, Brazil; 2 División de Investigaciones Epidemiológicas y Sociales, Instituto Mexicano de Psiquiatría (IMP), México, D.F., Mexico; 3 Department of Social Medicine Post-Graduate Program in Public Health, Health Sciences Center, Universidade Federal do Espírito Santo, Vitória, ES, Brazil; University of Stellenbosch, SOUTH AFRICA

## Abstract

**Background:**

Childhood adversities have been associated with a number of medical and psychiatric outcomes. However, the reported effects that specific childhood adversities have on suicidality vary across studies.

**Method:**

This was a cross-sectional, stratified, multistage area probability investigation of a general population in Brazil, designated the São Paulo Megacity Mental Health Survey. The World Mental Health Composite International Diagnostic Interview was applied in 5037 individuals ≥ 18 years of age, in order to assess 12 different adversities occurring during childhood and/or adolescence, as well as to look for associations between those adversities and subsequent suicidality in different age strata.

**Results:**

Over half of the respondents reported at least one childhood adversity. Only physical abuse was consistently associated with suicide attempts in all subsequent life stages (OR = 2.1). Among adults 20–29 years of age, the likelihood of a suicide attempt was correlated with parental divorce, whereas suicidal ideation was associated with prior sexual abuse. Among adults over 30 years of age, physical illness and economic adversity emerged as relevant childhood adversities associated with suicide attempts, whereas sexual abuse, family violence, and economic adversity were associated with suicidal ideation.

**Conclusion:**

Childhood adversities, especially physical abuse, are likely associated with unfavorable consequences in subsequent years. For suicidality across a lifespan, the role of different childhood adversities must be examined independently.

## Introduction

During the early developmental stages of life, individuals experience a constellation of negative life events, such as parental pathology, sexual abuse, and economic deprivation, which are known as childhood adversities. Previous studies have demonstrated an association between suicide attempts and exposure to childhood adversities, as well as demonstrating a dose-response gradient between the number of childhood adversities and the odds of suicidality [1, 2]. Most studies on childhood adversity have been conducted in high-income countries [1, 3–6]. One exception was a comprehensive study that examined the association between childhood adversity and suicidality in a cross-national sample, in high- and low-income countries [7]. In general, only a few studies have indicated a association between childhood adversity and subsequent suicidal behavior, and even fewer have taken into account short-term outcomes, occurring temporally close to a stressor, versus long-term, delayed outcomes of childhood adversities, in terms of their association with suicidality. Therefore, knowledge of the association between early adversities and suicide remains limited.

Rather than being rare, childhood adversities are pervasive events in epidemiologic samples, and their co-occurrence in the same individual seems to be the norm instead of the exception [8–10]. Such clustering of negative life events has been implicated in long-term medical outcomes (e.g., physical inactivity, severe obesity, hypertension, and headache) as well as psychiatric outcomes [3, 5, 11–14]. For instance, childhood adversities have been associated with a worse course of psychiatric disorders, such as depressive disorders [15], and with higher rates of suicidality [16]. Such outcomes have not been sufficiently investigated.

Suicide and self-harm behaviors are acknowledged as worldwide public health problems, calling for local policies to prevent their occurrence in communities. In Brazil, the situation is no different. Although, due to its relatively large population, the country presents a low overall annual age-adjusted suicide rate (6.0/100,000 population), it ranks eighth among all countries in terms of the total number of suicide deaths [17]. Regional variations in suicide rates have been reported in Brazil. For instance, over the last 20 years, the rates for the city and the state of São Paulo have both been approximately 4.5 suicides/100,000 population [18], which is slightly lower than that observed in the country as a whole.

The metropolitan area of São Paulo (MASP) presents a complex environment for studies on suicidal and violence-related behaviors. The MASP is the fourth largest metropolitan area in the world and is home to approximately 10% of the population of Brazil [19]. Uncontrolled urbanization and historically high social inequality in some deprived neighborhoods have contributed to high rates of violence and homicide [20, 21]. The exposure to increasing urban violence [22] could be compared to that experienced in countries where there are civil armed conflicts, such as Lebanon [23], or in others where violence is endemic, such as Nigeria and South Africa [24, 25]. The adverse consequences of such environments on public health need to be better understood.

Knowledge of the role of childhood adversities as precursors of suicidal cognitions and behaviors is needed in order to improve understanding of their temporal relationship with those factors [26, 27], as well as having implications for preventive interventions. Population-based studies provide an opportunity to perform a comprehensive examination of this under-researched topic in the general population. The aims of current study were to report the prevalence of childhood adversities in the MASP; to estimate the association between childhood adversities and suicidality; to determine the impact that the number of comorbid childhood adversities has on suicidality; and to depict the associations that childhood adversities have with suicidal behavior and cognitions occurring in individuals at various ages.

## Material and Methods

### Sampling

Designated the São Paulo Megacity Mental Health Survey, this was a cross-sectional investigation of a general population in Brazil. Using stratified, multistage area probability sampling, we selected a representative sample (*N* = 5037) of household residents ≥ 18 years of age in the MASP. Located in southeastern Brazil, the MASP comprises the city of São Paulo and 38 surrounding municipalities. At the time of data collection (May 2005 to May 2007), there were approximately 20 million inhabitants over the age of 18 in the area [28]. Detailed descriptions of the sampling procedure and weighting methods are presented elsewhere [29]. Table 1 shows the main demographic characteristics of the sample, which had a slight predominance of females (56.58%). Subjects 18–39 years of age accounted for 49.23% of the sample; 60.52% were married or cohabiting; and 60.15% were employed. The most common level of education (in 33.55%) was high-average (9–11 years of schooling), and 27.14% of the subjects reported a low-average family income.

**Table 1:** Descriptive characteristics of the participants of São Paulo Megacity Mental Health Survey (N = 5,037).

Variable	*n*	%
**Gender**		
Male	2,187	43.42
**Female**	**2850**	**56,58**
**Age, years**		
18–29	1287	25.55
30–39	1193	23.68
40–49	1090	21.64
50 or more	1467	29.12
**Education (years of schooling)**		
Low (0–4)	1344	26.68
Low-average (5–8)	1262	25.05
High-average (9–11)	1690	33.55
High (≥ 12)	741	14.71
**Family income**		
Low	1200	23.82
Low-average	1367	27.14
High-average	1212	24.06
High	1258	24.98
**Marital status**		
Married/cohabiting	3250	64.52
Previous married	894	17.75
Never married	893	17.73
**Employment status**		
Employed	3030	60.15
Student	56	1.11
Homemaker	836	16.60
Retired	494	9.81
Unemployed	621	12.33

### Diagnostic assessment

In order to generate diagnoses based on the criteria of the Diagnostic and Statistical Manual of Mental Disorders, Fourth Edition, we used the World Health Organization World Mental Health Composite International Diagnostic Interview (WMH-CIDI) [30], which is a fully structured interview applied by lay or professional examiners in face-to-face interviews. The WMH-CIDI is composed of two parts: part I comprises questions regarding sociodemographic characteristics, daily functioning, and physical morbidity, together with a core diagnostic assessment (e.g., for major depression, mania, panic disorder, substance use disorders, and suicidal behavior); and part II comprises questions regarding mental health risk factors, consequences and other correlates, together with assessments for additional disorders. Part II was administered to all respondents who met the lifetime criteria for any disorder in part I, as well as to those included in a probability subsample (*n* = 2942) intended to reduce the respondent burden and control the costs of the study. The WMH-CIDI allows the diagnosis of 20 mental disorders: major depressive disorder; bipolar I and II disorder; dysthymia; panic disorder; agoraphobia; social phobia; specific phobia; childhood separation anxiety disorder; adult separation anxiety disorder; generalized anxiety disorder; post-traumatic stress disorder; alcohol abuse; drug abuse; alcohol dependence; drug dependence; intermittent explosive disorder; oppositional-defiant disorder; conduct disorder; and attention-deficit/hyperactivity disorder. It also assesses the age at onset of each disorder, through a series of questions designed to improve accuracy of retrospective reports and avoid implausible response patterns [31, 32]. To avoid the recall bias that can occur among older respondents, oppositional-defiant disorder, conduct disorder, and attention-deficit/hyperactivity disorder were assessed only in those between 18 and 44 years of age. Sociodemographic variables assessed by the WMH-CIDI included age (in years), gender (male or female), years of schooling (0–4, 5–8, 9–11, or ≥ 12; referred to as low, low-average, high-average, and high levels of education, respectively), marital status (married/cohabiting, previously married, or never married), and family income (low, low-average, high-average, or high).

### Assessment of suicidality

The suicidality module of the WMH-CIDI was used in order to assess the lifetime occurrence of suicidal behaviors and cognitions, as well as the age at onset of suicidal ideation, suicide planning, and suicide attempts. For the purpose of our analyses, we considered four factors related to lifetime suicidality: suicidal ideation in the total sample; suicide attempts in the total sample; suicide planning among ideators; and suicide attempts among ideators.

### Assessment of childhood adversities

Childhood adversities, defined as those occurring before 18 years of age [33, 34], comprise 12 dichotomous variables that can be divided into four groups: parental maladjustment (mental illness, substance misuse, criminality, or family violence); maltreatment (physical abuse, sexual abuse, or neglect); loss of a biological or non-biological parent (through death, divorce, or other means); and other childhood adversities (life-threatening physical illness or family economic adversity). All childhood adversities were retrospectively assessed and coded using the criteria established by the World Mental Health Survey Initiative [10] in a dichotomous paradigm (yes/no) on whether or not the respondents had ever experienced a given childhood adversity.

We assessed childhood adversities using various strategies. Parental mental illness (depression, generalized anxiety disorder, panic disorder, or antisocial personality disorder) and parental substance misuse were assessed with the Family History Research Diagnostic Criteria Interview and its extensions [35, 36]. Parental criminality was assessed with measures created for the baseline US National Comorbidity Survey [5] and used in previous surveys [10, 34, 37]. Family violence—beating, slapping, hitting, pushing, grabbing, shoving, or throwing something at the child—perpetrated by the father or mother (biological parents, step-parents, or adoptive parents) or by anyone who nurtured the child, was assessed with a modified version of the Conflict Tactics Scale [38], as was physical abuse. Neglect was evaluated with questions commonly used in evaluating child welfare, regarding the frequency of the following: having inadequate supervision; having to do age-inappropriate tasks, jobs, or chores; and receiving inadequate food, clothing, or medical care [39]. Sexual abuse—including sexual intercourse, penetration with a finger or object, and sexual assault or molestation—was assessed with questions about repeated fondling, attempted rape, or rape. Sexual abuse was assessed in such a way that it remained unknown whether it had occurred within the family or not. However, on the basis of the findings of previous studies, it was assumed that if the experience had occurred one or two times, it was committed by a stranger, whereas if it occurred three or more times, it was committed by a family member [40]. Loss of a parent—through death, divorce (or separation), or parental or respondent absence for 6 or more months (because of overseas service in the armed forces, imprisonment, lengthy hospitalization, attending boarding school, or other reasons)—was assessed with the measures created for the baseline US National Comorbidity Survey [5] and used in previous surveys [10, 34, 37]. Economic adversity was also assessed with the measures created for the baseline US National Comorbidity Survey [5], and life-threatening childhood physical illness was assessed with a standard chronic conditions checklist [41].

### Statistical analysis

Because of the stratified, multistage area probability sampling design, the analytic approach included conventional methods for variance estimation with complex sample survey data. Weights were used to adjust for differences in within-household probability of selection and non-response, as well as to adjust for the differential sampling of WMH-CIDI part I respondents into WMH-CIDI part II. In a cross-classification of sociodemographic variables [29], a post-stratification weight was used in order to make the sample distribution comparable to the population distribution in the 2000 census. The Taylor series linearization method, implemented in the SUDAAN software package, version 8.0.1 (Research Triangle [42]), was used in order to generate the results of this complex sample design and to weigh and estimate standard errors for proportions of participants experiencing childhood adversity.

The 12-month prevalence of suicidal ideation, suicide planning, and suicide attempts was evaluated using cross-tabulations. The associations of potential correlates with suicide attempts and suicidal ideation (with or without planning or attempts) were estimated using bivariate and multivariate logistic regression analysis models. In the bivariate model, one adversity at a time was considered, whereas in the multivariate model, all adversities were considered at the same time. The 12-month prevalence of childhood adversities was also evaluated, for individuals and for the total sample. To facilitate the interpretation of the results, all logistic regression coefficients and 95% confidence intervals (CIs) were exponentiated and converted to odd ratios (ORs). To minimize the effects of outlier values, continuous variables were converted to categorical variables and the categories were collapsed when the ORs were not sufficiently different to stabilize the associations.

The associations between childhood adversities and suicidality were examined using discrete-time survival models [43], in which the unit of analysis was person-years, each year of life of each subject up to and including the year of onset of a disorder being treated as a separate observational record. The years prior to onset were coded as 0, the year of onset was coded as 1, and all subsequent years were excluded. In the subjects who had never been exposed to childhood adversity, all years of life up to the year of the interview were included. Excluding the person-years after the onset of the outcome from the data array allowed us to interpret the logistic regression coefficients as survival coefficients.

The bivariate and multivariate analyses to estimate the associations between childhood adversities and suicidality were performed step-by-step in order to evaluate the role of childhood adversities in situations of greater complexity. We then also analyzed the association between the number of childhood adversities and suicidality. In the multivariate model, we estimated the association between the type and number of childhood adversities and suicidality; the number and type of childhood adversities experienced by each respondent were included as dummy variables. Finally, we repeated the former analysis, although, in order to test long-term association between childhood adversity and suicidality at different ages, we tested also the association between type and number of childhood adversities and suicidality occurring in three different age categories (adolescence, early adulthood, and later adulthood). The analysis by age considered the influence that each type of adversity had on suicidality, stratified as early-onset (at 13–19 years of age), intermediate-onset (at 20–29 years of age), and late-onset (at ≥ 30 years of age), taking in account the interactions between those life stages for each respondent and each type of childhood adversity. All associations between adversities and suicidality were adjusted for sex, age, level of education, marital status, interactions between demographic variables, life course, and lifetime mental disorders, as well as for parental psychopathology (major depressive episodes, generalized anxiety disorder, panic disorder, substance use disorder, antisocial personality disorder, and suicidal behavior). The influence of lifetime mental disorders on suicidality was also examined, as was the interaction between sex and each type of adversity.

We estimated significance and standard errors with the Taylor series method [44], using the SUDAAN software to adjust for design effects. Wald chi-square tests based on design-corrected coefficient variance–covariance matrices were used in order to evaluate multivariate significance. All statistical tests were two-tailed, and values of *p* ≤ 0.05 were considered significant.

### Ethics statement

The study was approved by the Research Ethics Committee of the University of Sao Paulo School of Medicine *Hospital das Clínicas*. All participating respondents gave written informed consent and were assured that total confidentiality would be maintained.

## Results

### Prevalence of childhood adversities in the total sample

**Fig 1:**
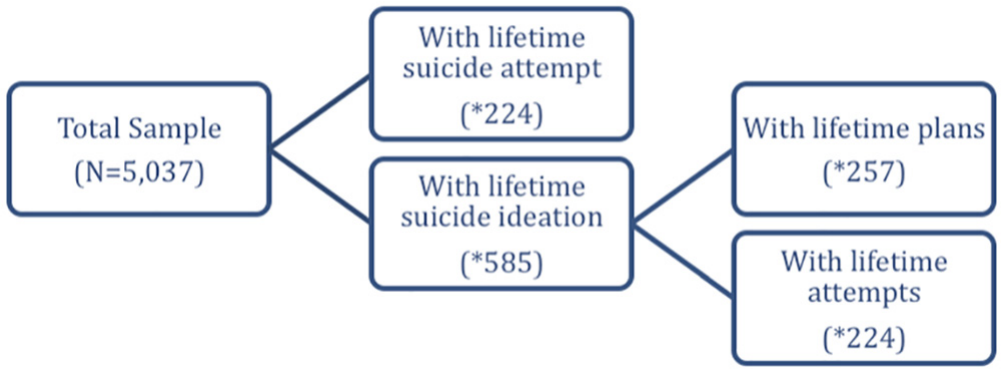
Schematic representation of the sample—Number of cases with the outcome variable; N represents the number of person-years.

A diagram of the suicidality data can be seen in Fig 1. Of the 5037 individuals evaluated, (53.6%) had experienced adversity at least once during childhood or adolescence. The most common childhood adversities were death of a parent (16.1%), and physical abuse in childhood (16.0%). Comorbidity was also found to be common and 48.4% of all respondents have experienced two or more adversities. That proportion varies according to the childhood adversity considered from 47.1% to 95.2%.

### Childhood adversities in individuals reporting suicidality

The prevalence of childhood adversities was higher among the individuals reporting suicidality (73.9%) than among the remaining respondents (53.6%). Among such individuals, the most common childhood adversities were physical abuse (frequency varying from 31.5% to 37.4%), and parental mental disorders (frequency varying from 22.9% to 31.4%), whereas the least common were physical illness (frequency varying from 2.0 to 3.0), and economic adversities (frequency varying from 2.6 to 3.0) (Table 2).

**Table 2:** Weighted prevalence and distribution of chronic childhood adversities in the total sample and among individuals with suicidality (N = 5,037).

	Total sample		Sample of individuals reporting suicidality			
	Prevalence of adversity	Among those with adversity: % with 2+ adversities	Total sample with suicidality		Among Ideators	
			adversities among	adversities among	adversities among	adversities among
Childhood adversity	% (SE)	% (SE)	Suicide Attempt % (SE)	Suicide Ideation % (SE)	Suicide Plan % (SE)	Suicide Attempt % (SE)
Physical Abuse	16.0 (1.1)	73.7 (3.3)	35.8 (4.5)	31.5 (2.9)	37.4 (3.8)	35.8 (4.5)
Sexual Abuse	0.7 (0.2)	95.2 (2.7)	4.3 (1.8)	2.1 (0.6)	3.9 (1.3)	4.3 (1.7)
Neglect	11.3 (0.9)	85.8 (2.2)	29.4 (4.4)	19.7 (2.2)	25.9 (3.5)	29.4 (4.4)
Parent Died	16.1 (1.0)	47.1 (4.2)	19.9 (3.6)	19.0 (2.7)	17.7 (2.8)	17.7 (2.8)
Parent Divorced	9.5 (0.7)	60.7 (4.3)	11.5 (2.9)	12.0 (1.8)	12.7 (2.5)	12.7 (2.5)
Other Parent Loss	8.4 (0.9)	73.8 (3.0)	14.2 (3.6)	12.6 (1.8)	17.9 (3.3)	17.9 (3.3)
Family Violence	12.1 (0.8)	87.5 (2.3)	28.0 (4.2)	23.6 (2.3)	28.8 (3.6)	28.8 (3.6)
Parent Mental Disorders	11.8 (0.9)	76.9 (2.5)	31.2 (4.5)	22.9 (2.4)	31.4 (3.8)	31.4 (3.8)
Parent Substance Use	9.7 (0.9)	78.3 (3.5)	21.1 (3.9)	14.5 (1.8)	14.4 (2.6)	14.4 (2.6)
Parent Criminal Behavior	3.6 (0.4)	89.1 (4.1)	7.1 (2.6)	6.8 (1.3)	9.0 (2.5)	9.0 (2.5)
Physical Illness	1.4 (0.4)	62.2 (13.1)	3.0 (2.0)	2.0 (0.7)	2.4 (1.9)	2.4 (1.9)
Economic Adversity	1.0 (0.2)	94.4 (2.9)	2.6 (1.4)	3.0 (1.0)	2.6 (1.3)	2.6 (1.3)
Any Adversity	53.6 (1.8)	48.4 (1.5)	80.1 (3.6)	73.9 (3.0)	80.8 (2.9)	80.1 (3.6)

### Bivariate and multivariate association between types of childhood adversity and suicidality

Table 3 shows that, in bivariate models, after adjustment for sociodemographic variables and parental psychopathology, five of the nine childhood adversities correlated with lifetime suicide attempts (OR varying from 1.1 to 8.5), whereas only physical abuse and physical illness correlated with lifetime suicidal ideation (OR varying from 1.0 to 6.6). Among suicide ideators, only neglect and loss of a parent to a cause other than death or divorce correlated with lifetime plans (OR varying from 1.0 to 3.6), and none of the childhood adversities correlated with suicide attempts.

**Table 3:** Bivariate and multivariate model for associations between child adversities and lifetime (LT) suicidality^1^.

	Total sample^2^^,^^3^				Among Ideators^4^^,^^5^			
	LT Attempts		LT Ideators		LT Plans		LT Attempts	
	Bivariate model	Multivariate model	Bivariate model	Multivariate model	Bivariate model	Multivariate model	Bivariate model	Multivariate model
Effect	OR (95% CI)	OR (95% CI)	OR (95% CI)	OR (95% CI)	OR (95% CI)	OR (95% CI)	OR (95% CI)	OR (95% CI)
Physical Abuse in childhood	2.4 (1.7–3.5)*	2.0 (1.4–2.9)*	2.1 (1.6–2.9)*	1.9 (1.5–2.6)*	1.2 (0.8–2.0)	1.0 (0.5–2.0)	1.2 (0.7–2.0)	1.2 (0.7–2.1)
Sexual Abuse in childhood	3.1 (1.1–8.5)*	2.1 (0.8–5.6)	2.0 (1.0–3.9)*	1.3 (0.7–2.4)	2.3 (0.6–8.4)	2.1 (0.6–6.7)	1.5 (0.3–7.2)	1.3 (0.3–6.4)
Neglect in childhood	1.8 (1.1–2.9)*	1.3 (0.8–2.1)	1.4 (1.0–2.0)	1.0 (0.7–1.5)	2.0 (1.2–3.4)*	1.7 (0.8–3.5)	1.1 (0.6–2.2)	1.2 (0.6–2.4)
Parent Died in childhood	1.1 (0.7–1.5)	1.0 (0.7–1.5)	1.2 (0.9–1.5)	1.1 (0.8–1.4)	1.3 (0.9–2.0)	1.4 (0.9–2.1)	0.7 (0.4–1.4)	0.8 (0.4–1.5)
Parent Divorced in childhood	1.4 (0.7–2.9)	1.3 (0.6–2.7)	1.3 (0.8–1.9)	1.1 (0.7–1.8)	1.6 (0.8–3.0)	1.4 (0.6–3.1)	1.5 (0.6–3.5)	1.5 (0.5–4.5)
Other Parent Loss in childhood	1.3 (0.6–2.7)	1.1 (0.6–2.3)	1.3 (0.8–2.2)	1.1 (0.7–1.8)	1.9 (1.0–3.6)*	1.7 (1.0–3.1)	0.7 (0.3–1.7)	0.7 (0.3–1.6)
Family Violence in childhood	1.8 (1.2–2.7)*	1.4 (0.9–2.1)	1.7 (1.3–2.4)*	1.4 (1.0–1.9)*	1.5 (0.7–3.2)	1.2 (0.6–2.7)	0.8 (0.5–1.4)	0.7 (0.4–1.4)
Physical Illness in childhood	3.1 (1.1–8.5)*	2.6 (1.0–6.8)*	1.6 (0.7–3.9)	1.4 (0.7–2.7)	0.8 (0.1–6.1)	0.6 (0.1–7.5)	1.1 (0.6–2.2)	1.4 (0.7–2.8)
Economic Adversity in childhood	2.4 (1.0–6.0)	2.0 (0.9–4.9)	3.7 (2.1–6.6)*	3.1 (1.8–5.6)*	1.4 (0.2–8.1)	0.9 (0.1–8.6)	1.3 (0.3–5.5)	0.9 (0.2–4.9)

*Significant at the .05 level, two-sided test

^1)^ Assessed in Part 2 sample due to having part 2 controls. Controls for the model include intervals (1–5 intervals), and also include significant variables from demographic, parent psychopathology models. Details in following footnotes.

^2)^ Models controls for intervals (1–5 intervals), demographics (sex, age, time-varying education, time-varying marriage), interaction between intervals (13–19,20–29,30+) and sex, age, education, marriage. For parent psychopathology, controlling for types of parental disorders (6 dummies, 1 for each disorder) and number of parent disorders (2+).

^3)^ Models controls for intervals (1–5 intervals), demographics (sex, age, time-varying education, time-varying marriage), interaction between intervals (13–19,20–29,30+) and sex, age, education, marriage. For parent psychopathology, controlling for types of parental disorders (6 dummies, 1 for each disorder) and number of parent disorders (2,3+).

^4)^ Models controls for intervals (1–5 intervals), demographics (sex, age, time-varying education, time-varying marriage), interaction between intervals (13–19,20–29,30+) and age, education. Parent psychopathology was not included due to insignificance in previous models.

^5)^ Models controls for intervals (1–5 intervals), demographics (sex, age, time-varying education, time-varying marriage), interaction between intervals (13–19,20–29,30+) and sex, age, education. Parent psychopathology was not included due to insignificance in previous models.

Table 3 also shows that, after adjustment for exposure to multiple adversities, few of the childhood adversities included in the multivariate models correlated with suicidality. Exceptions included physical abuse and physical illness, which retained the significance of their correlation with suicide attempts, despite showing lower ORs than those obtained in the bivariate models (OR = 2.0 and 2.6, respectively). Other exceptions were physical abuse (OR = 1.9), family violence (OR = 1.1), and economic adversity (OR = 3.1), which retained the significance of their correlation with suicide ideation on the total sample. Among the suicide ideators, none of the childhood adversities correlated with suicide planning or attempts.

### Bivariate association between the number childhood adversities and lifetime suicidality

Apropos of the association between the number of childhood adversities and suicidality (Table 4), there was a dose–response gradient between the number of childhood adversities and lifetime suicide attempts in the total sample for two or three childhood adversities. There was also a dose–response gradient between the number of childhood adversities and lifetime suicide ideation in the total sample, for one up to four or more childhood adversities (OR = 1.5–3.3). Among suicide ideators, there was no dose–response effect, although the presence of three childhood adversities presented an OR of 2.3 for those who were suicide planners.

**Table 4:** Associations between the number of child adversities and lifetime suicidality^1^,^†^.

	Total sample^2^^,^^3^				Among Ideators^4^^,^^5^			
	LT Attempts		LT Ideators		LT Plans		LT Attempts	
Number of child adversities	OR (95% CI)	Chi square	OR(95% CI)	Chi square	OR(95% CI)	Chi square	OR(95% CI)	Chi square
1	1.4 (0.7–2.5)		1.5 (1.1–2.1)*		1.4 (0.8–2.4)		0.8 (0.3–1.7)	
2	2.9 (1.7–4.8)*		2.1 (1.6–2.9)*		2.0 (0.9–4.8)		1.7 (0.7–4.5)	
3	3.0 (1.7–5.1)*	37.1 (< .001)*	2.4 (1.3–4.3)*		2.3 (1.1–5.1)*	5.2(0.16)	0.8 (0.4–1.6)	7.9(0.048)*
4 or more			3.3 (2.2–4.9)*	63.0 (< .001)*				

* Significant at the .05 level, two-sided test

^†^ All models were controlled for demographics (sex, age, time-varying education, and time-varying marriage), interaction between life course intervals (13–19, 20–29, 30+) and age, education, marriage.

^1)^ Assessed in Part 2 sample due to having part 2 controls. Controls for the model include int (1–5 intervals), and also include significant variables from demographic and parent psychopathology models, details in following footnotes.

^2)^ Models controls for intervals (1–5 intervals), countries, demographics (sex, age, time-varying education, time-varying marriage), interaction between intervals (13–19,20–29,30+) and sex, age, education, marriage. For parent psychopathology, controlling for types of parental disorders (6 dummies, 1 for each disorder) and number of parent disorders (2+).

^3)^ Models controls for intervals (1–5 intervals), countries, demographics (sex, age, time-varying education, time-varying marriage), interaction between intervals (13–19,20–29,30+) and sex, age, education, marriage. For parent psychopathology, controlling for types of parental disorders (6 dummies, 1 for each disorder) and number of parent disorders (2,3+).

^4)^ Models controls for intervals (1–5 intervals), countries, demographics (sex, age, time-varying education, time-varying marriage), interaction between intervals (13–19,20–29,30+) and age, education. Parent psychopathology was not included due to insignificance in previous models.

^5)^ Models controls for intervals (1–5 intervals), countries, demographics (sex, age, time-varying education, time-varying marriage), interaction between intervals (13–19,20–29,30+) and sex, age, education. Parent psychopathology was not included due to insignificance in previous models.

### Multivariate associations between type and number of childhood adversities and lifetime suicidality

Table 5 shows the results of the final multivariate model, which took into account the number and types of childhood adversities. For the total sample, physical abuse remained associated with suicide attempts (OR = 2.1) and with suicidal ideation (OR = 2.2), whereas economic adversity and family violence correlated with suicide ideation (OR = 3.8 and 1.7, respectively). Among the suicide ideators who were suicide planning, only the death of a parent and loss of a parent to a cause other than death or divorce remained associated with suicide planning (OR = 1.5 and 2.0, respectively). In this final multivariate model, the number of childhood adversities was not associated with suicidality.

**Table 5:** Final multivariate model for associations between child adversity and lifetime (LT) suicidality^1^.

	Total sample		Among ideators	
	LT Attempts^2^	LT Ideation^3^	LT Plans^4^	LT Attempts^5^
Effect	OR (95% CI)	OR (95% CI)	OR (95% CI)	OR (95% CI)
Physical Abuse in childhood	2.1 (1.2–3.7)*	2.2 (1.4–3.5)*	1.1 (0.5–2.3)	1.0 (0.5–2.3)
Sexual Abuse in childhood	2.2 (0.8–6.2)	1.6 (0.8–3.5)	2.2 (0.7–6.8)	1.3 (0.3–5.9)
Neglect in childhood	1.4 (0.7–2.6)	1.3 (0.8–2.1)	2.0 (0.9–4.6)	1.3 (0.5–3.1)
Parent Died in childhood	1.0 (0.7–1.7)	1.3 (0.9–1.8)	1.5 (1.0–2.4)*	0.8 (0.4–1.6)
Parent Divorced in childhood	1.3 (0.6–3.2)	1.3 (0.8–2.1)	1.6 (0.6–4.0)	1.4 (0.4–4.7)
Other Parent Loss in childhood	1.2 (0.5–2.9)	1.4 (0.8–2.4)	2.0 (1.0–4.2)*	0.7 (0.2–1.9)
Family Violence in childhood	1.5 (0.8–2.5)	1.7 (1.1–2.8)*	1.4 (0.6–3.1)	0.7 (0.3–1.7)
Physical Illness in childhood	2.6 (0.9–7.6)	1.6 (0.7–3.9)	0.7 (0.1–7.9)	1.3 (0.5–3.4)
Financial Adversity in childhood	2.0 (0.8–5.2)	3.8 (1.8–8.0)*	1.1 (0.1–9.8)	0.9 (0.2–5.4)
	**χ**^**2**^	**χ**^**2**^	**χ**^**2**^	**χ**^**2**^
group significance test for all types	24.6 (0.003)*	25.5 (0.002)*	15.2 (0.08)	10.1 (0.34)
significance test for difference between types	13.1 (0.11)	23.6 (0.003)*	13.2 (0.11)	10.4 (0.24)
2 CAs	1.3 (0.5–3.2)	0.8 (0.4–1.6)	1.1 (0.4–2.9)	2.0 (0.8–5.4)
3 CAs	0.8 (0.2–2.8)	0.6 (0.2–1.5)	0.6 (0.2–2.2)	0.9 (0.1–6.7)
4 or more CAs	-	0.4 (0.1–2.1)	-	-
	**χ**^**2**^	**χ**^**2**^	**χ**^**2**^	**χ**^**2**^
	4.5 (0.10)	2.9 (0.40)	1.4 (0.49)	5.7 (0.06)

*Significant at the .05 level, two-sided test

^1)^ Assessed in Part 2 sample due to having part 2 controls. Controls for the model include intervals (1–5 intervals), and also include significant variables from demographic and parent psychopathology models, details in following footnotes.

^2)^ Models controls for intervals (1–5 intervals), demographics (sex, age, time-varying education, time-varying marriage), interaction between intervals (13–19,20–29,30+) and sex, age, education, marriage. For parent psychopathology, controlling for types of parental disorders (6 dummies, 1 for each disorder) and number of parent disorders (2+).

^3)^ Models controls for intervals (1–5 intervals), demographics (sex, age, time-varying education, time-varying marriage), interaction between intervals (13–19,20–29,30+) and sex, age, education, marriage. For parent psychopathology, controlling for types of parental disorders (6 dummies, 1 for each disorder) and number of parent disorders (2,3+).

^4)^ Models controls for intervals (1–5 intervals), demographics (sex, age, time-varying education, time-varying marriage), interaction between intervals (13–19,20–29,30+) and age, education. Parent psychopathology was not included due to insignificance in previous models.

^5)^ Models controls for intervals (1–5 intervals), demographics (sex, age, time-varying education, time-varying marriage), interaction between intervals (13–19,20–29,30+) and sex, age, education. Parent psychopathology was not included due to insignificance in previous models.

### Associations between the types of childhood adversity and lifetime suicidality over the life course

Table 6 shows additional multivariate analyses of the associations between adversities occurring during childhood and adolescence and subsequent suicidality throughout each period of life: adolescence, early adulthood, and later adulthood. The multivariate models take into account the type and the number of childhood adversities. The number of childhood adversities was excluded from the age-specific models.

**Table 6:** Multivariate model for associations between child adversity and lifetime (LT) suicidality during teen (13–19 y.o.), early adult (20–20 y.o.) and later adult years (30 or more y.o)^1^.

	Teen years (13–19 y.o.)		Early adulthood (20–29 y.o.)			Later adulthood (30 or more y.o)		
	Total sample		Total sample	Ideators		Total sample		Ideators
	LT Attempts^2^	LT Ideation^3^	LT Attempts^2^	LT Plans^4^	LT Attempts^5^	LT Attempts^2^	LT Ideators^3^	LT Plans^4^
Effect	OR (95% CI)	OR (95% CI)	OR(95% CI)	OR(95% CI)	OR(95% CI)	OR(95% CI)	OR(95% CI)	OR(95% CI)
Physical Abuse	2.5* (1.1–5.7)*	2.4* (1.3–4.3)*	1.8 (0.8–4.3)	0.9 (0.3–2.7)	1.0 (0.3–3.1)	2.0 (0.6–6.7)	2.2 (0.9–5.2)	0.7 (0.1–3.3)
Sexual Abuse	1.6 (0.3–8.5)	0.9 (0.2–3.4)	0.6 (0.1–4.5)	3.7 (1.0–14.2)*	1.0 (0.1–11.0)	5.0 (0.9–28.2)	5.5 (1.9–16.3)*	3.4 (0.4–28.2)
Neglect	1.2 (0.4–3.7)	0.9 (0.4–2.2)	1.4 (0.5–3.8)	1.4 (0.4–5.0)	1.1 (0.3–3.3)	1.8 (0.4–8.1)	1.5 (0.7–3.4)	0.9 (0.2–3.6)
Parent Died	0.8 (0.4–1.7)	1.0 (0.6–1.9)	1.8 (0.7–4.6)	1.5 (0.7–3.3)	1.8 (0.7–4.5)	0.8 (0.3–2.5)	1.6 (0.7–3.6)	0.5 (0.2–1.3)
Parent Divorced	0.7 (0.2–2.3)	1.0 (0.5–1.9)	2.8 (1.0–8.1)*	2.0 (0.6–7.4)	3.1 (0.8–11.9)	0.3 (0.0–2.1)	1.1 (0.4–2.8)	0.2 (0.0–2.3)
Other Parent Loss	1.7 (0.5–6.0)	1.6 (0.9–3.0)	0.9 (0.2–3.5)	2.5 (0.9–6.4)	0.4 (0.1–1.9)	1.2 (0.6–2.5)	1.2 (0.5–2.7)	0.5 (0.2–1.3)
Family Violence	1.3 (0.7–2.4)	1.6 (0.9–2.9)	1.6 (0.6–4.0)	1.5 (0.5–5.2)	1.0 (0.4–2.9)	1.5 (0.6–4.0)	2.1 (1.0–4.6)*	0.2 (0.0–0.7)*
Physical Illness	3.5 (0.4–29.3)	2.2 (0.7–7.0)	0.0* (0.0–0.0)*	0.2 (0.0–2.0)	0.0 (0.0–0.0)*	7.1* (1.9–26.0)*	1.1 (0.2–8.3)	0.9 (0.1–8.2)
Economic Adversity	1.5 (0.2–10.8)	2.6 (0.5–14.0)	0.6 (0.1–5.5)	0.3 (0.0–8.1)	0.6 (0.0–8.9)	5.7* (1.3–25.7)*	9.8 (3.5–27.9)*	0.8 (0.1–8.1)
	**χ**^**2**^	**χ**^**2**^	**χ**^**2**^	**χ**^**2**^	**χ**^**2**^	**χ**^**2**^	**χ**^**2**^	**χ**^**2**^
group significance test for all types	31.1 (<0.001)*	83.7 (<0.001)*	1671.2 (<0.001)*	27.4 (0.001)*	576.2 (<0.001)*	39.1 (<0.001)*	58.0 (<0.001)*	12.9 (0.17)
significance test for difference between types	20.1 (0.010)*	21.4 (0.006)*	1427.0 (<0.001)*	23.7 (0.003)*	542.4 (<0.001)*	33.2 (<0.001)*	47.5 (<0.001)*	11.8 (0.16)
**Number of CAs**								
2 CAs	1.0 (0.3–3.1)	0.6 (0.3–1.4)	1.9 (0.6–6.1)	1.6 (0.4–5.7)	2.4 (0.7–8.8)	1.2 (0.3–4.5)	0.7 (0.3–1.5)	3.1 (0.8–11.7)
3 CAs	0.7 (0.1–4.2)	1.1 (0.4–2.8)	0.8 (0.1–4.8)	0.5 (0.1–5.2)	0.6 (0.1–5.9)	0.9 (0.1–14.1)	0.5 (0.1–1.8)	12.4 (1.6–94.5)*
4 or more CAs	-	0.7 (0.1–5.1)	-	-	-		0.2 (0.0–1.5)	
	**χ**^**2**^	**χ**^**2**^	**χ**^**2**^	**χ**^**2**^	**χ**^**2**^	**χ**^**2**^	**χ**^**2**^	**χ**^**2**^
	0.6 (0.74)	3.2 (0.36)	5.2 (0.07)	3.4 (0.18)	8.6 (0.013)*	0.2 (0.90)	3.1 (0.38)	6.6 (0.036)*

*Significant at the .05 level, two-sided test

^1)^ Assessed in Part 2 sample due to having part 2 controls. Controls for the model include intervals (1–5 intervals), and also include significant variables from demographic and parent psychopathology models, details in following footnotes.

^2)^ Models controls for intervals (1–5 intervals), demographics (sex, age, time-varying education, time-varying marriage), interaction between intervals (13–19,20–29,30+) and sex, age, education, marriage. For parent psychopathology, controlling for types of parental disorders (6 dummies, 1 for each disorder) and number of parent disorders (2+).

^3)^ Models controls for intervals (1–5 intervals), demographics (sex, age, time-varying education, time-varying marriage), interaction between intervals (13–19,20–29,30+) and sex, age, education, marriage. For parent psychopathology, controlling for types of parental disorders (6 dummies, 1 for each disorder) and number of parent disorders (2,3+).

For suicidality occurring during adolescence (13–19 years of age), only reports of earlier physical abuse were relevant. In total sample, physical abuse correlated with suicide attempts (OR = 2.5) and with suicidal ideation (OR = 2.4). Among the suicide ideators, none of the types of childhood adversity evaluated correlated with suicide planning or attempts.

For suicidality occurring during early adulthood (20–29 years of age), only parental divorce in childhood or adolescence correlated with suicide attempts (OR = 2.8). Among the suicide ideators in that age group, the likelihood of suicidal plans among ideators correlated with early sexual abuse (OR = 3.7).

For suicidality occurring during later adulthood (≥ 30 years of age), suicide attempts correlated with physical illness during childhood or adolescence (OR = 7.1) and with economic adversity (OR = 5.7). Likewise, suicidal ideation correlated with sexual abuse (OR = 5.5), family violence (OR = 2.1), and economic adversity (OR = 9.8). Among the suicide ideators in that age group who engaged in suicide planning, only family violence correlated with suicidality (OR = 0.2).

## Discussion

### Childhood adversities

Childhood adversities occur commonly in the general population, and more than 50% of the individuals in our sample had experienced at least one childhood adversity. Among the subjects reporting suicidality, the lifetime occurrence of at least one childhood adversity was even higher (approximately 80%). Despite their high prevalence, most childhood adversities did not correlate with lifetime suicidality when the analysis was adjusted for the type and number of adversities. In the total sample, physical abuse during childhood emerged as the childhood adversity most consistently associated with suicide attempts and suicidal ideation. These results underscore the need to avoid violent disciplinary methods, as a primary measure for the prevention of suicidal behavior.

The prevalence of childhood adversities in the MASP (53.6%) is higher than that reported for high-, high-middle-, and lower-/low-income countries (38.4%, 38.9%, and 39.1%, respectively), as well as being higher than that calculated from the pooled data of a cross-national sample involving 21 countries (38.8%) [10]. The prevalence of individual childhood adversities varies considerably across samples, and cultural diversity might play a role in explaining the differences between countries. For example, the prevalence of childhood adversities in Brazil is similar to that reported for Mexico (54.5%) [37], but not to that reported for China (31.0%) [45], despite the fact that the three countries present similar levels of socioeconomic development. However, Brazil and Mexico are much more culturally related and have comparable levels of violence (neglect, physical abuse, and family violence).

In general, the frequency of childhood adversities appears to be directly proportional to the severity of suicidal behavior and cognitions. Comparing our data with those obtained for a worldwide cross-national sample [7], we found that, among the respondents reporting lifetime suicide attempts, the prevalence of physical abuse during childhood or adolescence was higher (38.5% vs. 29.3%), whereas that of a history of sexual abuse was lower (4.3% vs. 14.5%).

### Suicidal behavior

For our total sample, bivariate models showed that most childhood adversities correlated with suicide attempts later in life, the exceptions being those related to interpersonal loss (loss of a parent to death, divorce, or another cause) and economic adversity; only a few childhood adversities were found to correlate with suicidal ideation. However, unlike what has been reported in some previous studies [1, 2], many of those associations failed to retain their significance after we adjusted for the type and number of childhood adversities. This loss of association seen in the final multivariate model suggests that other factors, such as personality dimensions and psychiatric disorders, have a mediating and/or moderating effect on suicidality [46–48], attenuating the correlation between childhood adversity and suicidality. Similar results were obtained in a worldwide cross-national survey [7], as well as in studies conducted in underdeveloped countries such as South Africa and Nigeria [49] [50].

In our final models, physical abuse emerged as the childhood adversity most strongly associated with suicide attempts and suicidal ideation. That is in agreement with the findings of various cross-sectional studies [7, 49–51], as well as with those of a three-year longitudinal follow-up study [4]. It is noteworthy that, in our sample, sexual abuse was not associated with suicidality, although it has been associated with suicide attempts (impulsive or not) in other samples [7, 49–51]. That discrepancy could be attributed to the fact that the prevalence of sexual abuse in our sample was relatively low (0.7%).

When we evaluated suicide ideators separately, we found that none of the childhood adversities evaluated correlated with suicide attempts, even in the bivariate model, although the death of a parent and the loss of a parent to a cause other than death or divorce were significantly associated with the advent of suicide planning. This correlational profile differs across studies. In the previously cited worldwide cross-national survey [7], sexual abuse was associated with suicide attempts among the suicide ideators. However, in the study conducted in South Africa [49], such an association was observed only for physical abuse and not for sexual abuse.

Looking to the age of suicidality, in the present study, only physical abuse was ultimately associated with suicidality (suicide attempts) occurring during adolescence, a profile different from those described in the two other studies that analyzed such associations [7, 49]. For suicidality during young adulthood, we found that parental divorce was associated with almost three times the risk of a suicide attempt. Although physical abuse did not correlate with suicidality in that age group, we found that sexual abuse during childhood or adolescence results in a nearly four-fold increase in the likelihood of a suicide ideator presenting a plan during young adulthood. Among individuals in later adulthood, sexual abuse appears to be associated with suicidal ideation, indicating that this is a protracted association. It is of note that, despite their low prevalence in our sample, economic adversity and physical illness were also associated with suicide attempts later in life, underscoring the need to investigate such events even in older individuals.

The MASP presents a high prevalence of and a generalized exposure to violence. That environmental context, which also includes social inequality, a high level of social isolation, changes in interpersonal relationships, and the dissolution of the nuclear family, propitiates greater exposure to adversities in early childhood. Remarkably, the MASP nevertheless presents low rates of suicide. Mexico City, another megacity, presents a similarly high rate of childhood adversities and also has a low suicide rate [17, 37]. One possible explanation for this can be found in the observations made in 1881 by Morselli [52], later corroborated by Durkheim [53], that suicide rates are lower during periods of social turbulence, as documented by Oron Ostre [54], who also noted that those rates continue to decline in proportion to the duration of the crises. Morselli and Durkheim both proposed that, in such periods, people tend to join forces in order to struggle against an “external” threat [53, 54]. Although Durkheim refers to foreign enemies in his theory [53], people living in endemic poverty and violent communities might act in response to internal threats as other people do in response to wars. In fact, it has been observed that, in most cases, people living in poor areas, such as slums (or *favelas*, as they are referred to in Brazil) present a social cohesion and a sense of community that create a sensation of loyalty to and identification with their place of living [55]. They live with dichotomous interchangeable feelings about their role in the social fabric of the city—an “us” versus “them” mentality [56]. That sense of cohesion and belonging to a small community could serve as buffer against violence and adversities. Although it has yet to be tested, this urban social phenomenon might be also present in other countries with high rates of violence, such as Colombia, Mexico, Nigeria, South Africa, and Honduras, all of which also have low suicide rates [17].

### Implications

The fact that physical abuse was the childhood adversity most strongly associated with suicidality in the present study is remarkable because it is a modifiable factor, whereas other childhood adversities, such as the death of a parent and physical illness, are not. A Brazilian federal law (Law no. 13.010/2014), popularly known as the *Lei da Palmada* (“Anti-Spanking Law”) [57], which went into effect in 2014, can certainly be seen as a primary preventive measure. The law prohibits the use of corporal punishment, as well as the use of cruel or degrading treatment as a way of correction, discipline, or education, of children and adolescents. However, enforcement of that law will face several cultural obstacles. In an online survey, 75% of Brazilian parents stated that they believe corporal punishment is an effective disciplinary method [58].

### Limitations

Our results should be interpreted bearing in mind certain limitations. An interview bias could have occurred, because some respondents might have been reluctant to report, in face-to-face interviews, some facts, events, or memories that they considered painful, uncomfortable, humiliating, shameful, and/or embarrassing. One example of such bias could be the low prevalence of sexual abuse, which suggests under-reporting. In the present study, the rates of sexual abuse were low and were comparable to those reported in studies conducted in South Africa (0.7–2.1%), Nigeria (1.4 to 4.8%), Lebanon (0.4%), and Japan (0.5%) [49, 50, 59, 60], all of which used methodologies analogous to that used here. That might have resulted in an underestimation of the association between childhood adversities and suicidality. In addition, because the data were collected retrospectively, the prevalence of childhood adversity might have been underestimated in some cases, due to a recall bias [61, 62]. The same bias could also apply to suicidality [34]. Another possible limitation is the fact that we used a 12-item questionnaire to identify childhood adversities, and some individuals could have experienced childhood adversities that were not included in the list. Furthermore, the fact that the timing, sequencing, persistence, recurrence, or severity of individual childhood adversities were not evaluated makes it possible that childhood adversities could have occurred after a suicidal behavior. The use of multivariate models with several estimates of the odds of various exposures and outcomes can increase the possibility of having statistically significant associations in different stages of life due only to chance. Because suicidality is not a common outcome, the statistical power of association between early childhood adversities and different periods of life might have been reduced due to small size of the sample of individuals developing suicidality. Larger confidence intervals support the likelihood of association by chance. Therefore, there is a need for further studies addressing this issue in samples that are more inclusive. Finally, genetic, prenatal, and perinatal precursors were not taken in account in our analysis. Despite these limitations, our findings allowed us to depict the role that childhood adversities play in the onset of suicidality.

## Conclusions

Childhood adversities, especially physical abuse, are important events with potential long-term consequences. They should therefore be systematically investigated in all individuals seeking mental health care, especially those with a history of suicidal behavior or cognitions. Nevertheless, their effects on suicidality have to be seen in the context of other risk factors for suicidal behavior. Future studies might investigate and depict the pathway linking childhood adversities with suicidality.

Although some childhood adversities have been recognized as being associated with suicidality later in life, the roles played by the different types of childhood adversities vary in magnitude, leading to diverse outcomes and different levels of severity. Our findings underscore the idea that violent physical abuse is a potentially modifiable childhood adversity, rooted in Brazilian culture, that should not be used as a punitive measure in the education of children. Appropriate instruction of parental and teaching skills might diminish the rates of suicidal behaviors in the public health domain.
